# Unique molecular features of the HIV-1 subtype C enhancer and core promoter and their influence on the viral gene expression

**DOI:** 10.1186/1742-4690-10-S1-P94

**Published:** 2013-09-19

**Authors:** Anjali Verma, Pavithra Rajgopalan, Rishikesh Lotke, Saihitha Veerapaneni, Mahesh Bachu, Udaykumar Ranga

**Affiliations:** 1 Molecular Biology and Genetics Unit, Jawaharlal Nehru Centre for Advanced Scientific Research, Bangalore, Karnataka, India

## Background

The HIV-1 Subtype C, among other viral subtypes, is responsible for nearly 50% of the global infections including approximately 99% of the infections in India (UNAIDS 2012). HIV-1 subtype C is characterized by the presence of three NF-κB binding sites in the enhancer as opposed to one or two such elements in other viral subtypes. Additionally, NF-κB site proximal to the Sp1 motifs, referred to here as the C-κB site, is genetically distinct from the other two canonical NF-κB sites (H-κB sites). Furthermore, the Sp1III site proximal to the C-κB motif also demonstrates subtype-specific genetic variations. Here, we examined the functional significance of the two unique regulatory elements, the C-κB and the Sp1III motifs, for gene expression regulation from the viral promoter (Perkins *et al.* 1997).

## Methods and results

Using several panels of reporter vectors expressing luciferase under the control of variant viral promoters, we examined gene expression in different mammalian cells under diverse activation conditions. The data confirm that the proximity and orientation of two sites, the C-κB and Sp1III motifs, are critical for optimal gene expression from the viral promoter. We found that a functional cooperation among the three NF-κB sites is necessary for maximal promoter activity. Collectively, the data appeared to suggest that in addition to conferring a quantitative gain-of-function on the viral promoter, the presence of C-κB site may have catalyzed associated genetic variation in the proximal Sp1III site through a positive selection pressure. To understand the functional association between these two sites under more relevant conditions, we constructed viral promoters consisting of only the C-κB and the Sp1III sites, in the absence of other NF-κB and Sp1 sites. The subtype C specific Sp1III site showed higher magnitude of gene expression with the H- but not with the C-κB site. Experiments, using infectious viruses and the chromatin immunoprecipitation analysis, have also suggested the functional association between the two sites. In gel-shift assays the C-κB site recruited the p50 and p65 hetero-duplexes at a level comparable to that of the H-κB site. Importantly, given that the C-κB site is a G/C-centric motif, we asked and found that the C-κB site is responsive to the p52:Bcl3 complex in contrast to the A/T-centric H-κB site known to be more responsive to the p50:p65 complex promoter.

## Conclusions

Collectively our data are suggestive of co-evolution of the two unique regulatory elements, the C-κB and the C-Sp1III motifs, that confer qualitative gain-of-function on the subtype C promoter thus probably playing a critical role towards the global predominance of the viral subtype. We further hypothesize that the C-κB site being a G/C-centric motif, in combination with the two upstream H-κB sites, confers a sustained and prolonged gene expression on HIV-1 subtype C viral promoter.

